# Effect of a Run‐In Period on Estimated Treatment Effects in Cardiovascular Randomized Clinical Trials: A Meta‐Analytic Review

**DOI:** 10.1161/JAHA.121.023061

**Published:** 2022-10-17

**Authors:** Robert P. Murphy, Martin J. O’Donnell, Aoife Nolan, Emer McGrath, Aengus O’Conghaile, John Ferguson, Alberto Alvarez‐Iglesias, Maria Costello, Elaine Loughlin, Catriona Reddin, Sarah Ruttledge, Sarah Gorey, Diarmaid Hughes, Andrew Smyth, Michelle Canavan, Conor Judge

**Affiliations:** ^1^ Health Research Board Clinical Research Facility‐Galway National University of Ireland Galway Galway Ireland; ^2^ Department of Neurology Harvard Medical School Boston MA; ^3^ Department of Psychiatry National University of Ireland Galway Galway Ireland; ^4^ Translational Medical Device Lab National University of Ireland Galway Galway Ireland; ^5^ Wellcome Trust – Health Research Board Irish Clinical Academic Training Galway Ireland

**Keywords:** cardiovascular prevention, meta‐analysis, run‐in, trial methodology, Clinical Studies, Vascular Disease

## Abstract

**Background:**

A run‐in period may increase adherence to intervention and reduce loss to follow‐up. Whether use of a run‐in period affects the magnitude of treatment effects is unknown.

**Methods and Results:**

We conducted a meta‐analysis comparing treatment effects from 11 systematic reviews of cardiovascular prevention trials using a run‐in period with matched trials not using a run‐in period. We matched run‐in with non–run‐in trials by population, intervention, control, and outcome. We calculated a ratio of relative risks (RRRs) using a random‐effects meta‐analysis. Our primary outcome was a composite of cardiovascular events, and the primary analysis was a matched comparison of clinical trials using a run‐in period versus without a run‐in period. We identified 66 run‐in trials and 111 non–run‐in trials (n=668 901). On meta‐analysis there was no statistically significant difference in the magnitude of treatment effect between run‐in trials (relative risk [RR], 0.83 [95% CI, 0.80–0.87]) compared with non–run‐in trials (RR, 0.88 [95% CI, 0.84–0.91]; RRR, 0.95 [95% CI, 0.90–1.01]). There was no significant difference in the RRR for secondary outcomes of all‐cause mortality (RRR, 0.97 [95% CI, 0.91–1.03]) or medication discontinuation because of adverse events (RRR, 1.05 [95% CI, 0.85–1.21]). Post hoc exploratory univariate meta‐regression showed that on average a run‐in period is associated with a statistically significant difference in treatment effect (RRR, 0.94 [95% CI, 0.90–0.99]) for cardiovascular composite outcome, but this was not statistically significant on multivariable meta‐regression analysis (RRR, 0.95 [95% CI, 0.90–1.0]).

**Conclusions:**

The use of a run‐in period was not associated with a difference in the magnitude of treatment effect among cardiovascular prevention trials.

**Nonstandard Abbreviations and Acronyms**
RRR: ratio of relative risk
UL: upper bound of CI

**Clinical Perspective**

**What Is New?**

- A run‐in period is used in randomized clinical trials to increase adherence to the intervention and reduce loss to follow‐up in clinical trials.
- It is not known whether the use of a run‐in period affects the magnitude of treatment effects in cardiovascular prevention trials.
- In this meta‐analysis of cardiovascular prevention clinical trials, differences in treatment effects for cardiovascular composite outcomes between trials with run‐in periods (relative risk [RR], 0.83 [95% CI, 0.80–0.87]) and matched trials with non–run‐in periods (RR, 0.88 [95% CI, 0.84–0.91]) were not statistically significant (ratio of relative risk, 0.95 [95% CI, 0.90–1.01]).

**What Are the Clinical Implications?**

- Our analysis reports that the use of a run‐in period, compared with matched trials that did not use a run‐in period, was not significantly associated with a difference in treatment estimates in cardiovascular prevention randomized clinical trials.

A prerandomization run‐in period is intended to improve the precision of treatment‐effect estimates by excluding participants who are nonadherent with study interventions or trial protocols.^1^, ^2^, ^3^, ^4^, ^5^ During a run‐in period, participants receive an intervention (either active or placebo), and only those deemed adherent and tolerant of the intervention during the run‐in period are randomly assigned in the trial. The main proposed advantage of a run‐in period is improved average drug adherence rates of randomly assigned participants and improved rates of follow‐up.^6^ However, if populations who are nonadherent with medications are also those at higher cardiovascular risk (eg, higher prevalence of comorbidities), use of a run‐in period may result in a trial population with lower event rates.^7^

An unproven concern of run‐in trials, given the systematic exclusion of nonadherent individuals, is the introduction of a selection bias, thereby affecting the external validity of the trial.^8^ This issue is most relevant to phase III randomized clinical trials where analyses are based on the intention‐to‐treat principle, in which the purpose is to represent summary treatment effects for a general population, including those who are nonadherent with the trial intervention. As such, one may speculate that the treatment effects reported in run‐in trials may be closer to an “on‐treatment” analysis than an intention‐to‐treat analysis given that it is expected to increase the proportion adherent with treatment. Moreover, if participants who fail the run‐in period are systematically different from those who succeed the run‐in period, especially if differences relate to treatment efficacy, the estimates of treatment efficacy and safety may also differ between run‐in and non–run‐in trials. To date, evidence to support or refute differences in treatment effect estimates is lacking despite the widespread use of run‐in periods.^9^, ^10^ It has also not been established whether the proposed benefits of run‐in, that is, improved adherence with the intervention and trial protocols, are substantiated in clinical practice. Employing a run‐in period increases the complexity of randomized clinical trials, adds more burden to study participants and researchers, and may increase trial costs.^11^ Accordingly, it is necessary to confirm the proposed benefits of run‐in periods and reliably exclude any association with biased treatment estimates.

In this study, we evaluated whether the use of a run‐in period in cardiovascular prevention trials is associated with differences in relative treatment effects and the rates of adverse events resulting in drug discontinuation, cardiovascular events, mortality, and loss to follow‐up.

## METHODS

### Data Sources

The data that support the findings of this study are available from the corresponding author upon reasonable request. A protocol detailing the design and methods of the current meta‐analysis has been published previously.^12^ In summary, we identified 11 systematic reviews published between 2010 and 2019 that reported both primary and secondary prevention trials of effective therapies for cardiovascular prevention, namely, antihypertensive, lipid‐lowering, and glucose‐lowering medications^13^, ^14^, ^15^, ^16^, ^17^, ^18^, ^19^, ^20^, ^21^, ^22^, ^23^ (Figure S1; Table S1). We selected systematic reviews of proven cardiovascular prevention benefit where the overall summary estimate of the meta‐analyses was significant to test the hypothesis that the run‐in trial might bias estimates of efficacy. The strategy of sourcing trials for inclusion from a range of high‐quality published systematic reviews allowed us to reduce research waste.^24^ We included all phase III randomized clinical trials from these systematic reviews where an effective medicinal product for cardiovascular prevention (ie recommended by current cardiovascular prevention guidelines) was compared with a placebo or standard‐of‐care control. Given that none of these published meta‐analyses selected trials based on run‐in status, we considered this approach to be associated with a lower risk of selection bias. We conducted primary data extraction independently from the primary trial report with data double checked by a second independent researcher. Any discrepancies were reviewed by both reviewers and resolved by consensus of the data extraction team, or if required, review with a senior author.

### Matching Process

We integrated a methodological approach based on previous meta‐analyses evaluating the effect of loss to follow‐up and early trial stopping rules on effect estimates of interventions in randomized clinical trials.^25^, ^26^

We completed the matching of run‐in trials with non–run‐in trials using a 3‐step approach. In step 1, we matched run‐in and non–run‐in trials by intervention, as we considered matching on drug class to be an essential criterion (eg, match angiotensin‐converting enzyme inhibitor with angiotensin‐converting enzyme inhibitor). Therefore, a mandatory matching criterion was an exact match for intervention (ie, needed to be the within the same drug class). Following step 1, we generated a score for each potential run‐in/non–run‐in trial pairing based on similarity of population, control, and outcome. A score of 0 was defined as not a match, 1 was defined as an acceptable match, 2 was defined as a close match, and 3 was defined as an exact match based on prespecified criteria (Table S2). In step 2, we included all potential matches from step 1 and applied a population score criterion of ≥1 (ie, minimum acceptable match). Therefore, following step 2, all potential matches had a minimum score of 4. Following this step, we had 1359 unique potential matches between run‐in and non–run‐in trials. In step 3, we assigned a matching score for all potential matched trial pairs, ranging from a minimum of 4 (a score of “acceptable match” in each population, intervention, control, and outcome domain) to a maximum of 12 (a score of “exact match” in each population, intervention, control, and outcome domain; Figure S2).

### Statistical Analysis

For each run‐in and non–run‐in trial, we calculated the individual trial relative risk (RR) for each outcome measure. If multiple non–run‐in trials matched to a single run‐in trial, we performed a random‐effects meta‐analysis of the non–run‐in trials to calculate the summary estimate to give a single non–run‐in RR to compare with the run‐in trial. This meant that for each matched comparison, a run‐in trial RR could either be matched against a single non–run‐in trial RR or a meta‐analysis of multiple non–run‐in RRs. Then for each matched comparison, we calculated a ratio of RRs (RRRs) and 95% CI by subtracting the log(non–run‐in trial RR) from the log(run‐in trial RR) with SEs calculated as the square root of the following: SE(Run‐in RR)^2^+SE(Non–run‐in RR)^2^. We conducted a random‐effects meta‐analysis of the RRRs between run‐in and matched non–run‐in trials to obtain a summary estimate of the RRRs. A ratio <1 indicates a greater treatment effect in the run‐in trials (compared with matched non–run‐in trials). We used a random‐effects model because run‐in trials were matched to variable numbers of non–run‐in trials, and we considered this approach to best represent the “average” log‐RR in this setting. We used random‐effects meta‐analysis because we considered that the true effect sizes being combined were exchangeable. ^27^ In addition, we performed sensitivity analysis using fixed‐effects meta‐analysis to ensure consistent findings regardless of the exact form of meta‐analysis used. We used Cochran’s Q test to test for heterogeneity and the I^2^ statistic to estimate the percentage of variability.

Our primary analysis was a comparison of the RR reported in run‐in trials compared with matched non–run‐in trials for the primary outcome (cardiovascular composite) and was confined to “best‐matched” trials (ie, maximum population, intervention, control, and outcome matched score) for this outcome. Non–run‐in trials could only be matched once to prevent trials with large treatment effects biasing the estimate. For a non–run‐in trial prematched to several run‐in trials, we selected the run‐in and non–run‐in pair with the largest population, intervention, control, and outcome score and run‐in trial sample size (Figure S3). We completed analyses of secondary clinical outcomes, all‐cause mortality, adverse events leading to medication discontinuation, nonfatal myocardial infarction, nonfatal stroke, and loss to follow‐up.

Sensitivity analyses were conducted where non–run‐in trials were permitted to match multiple times to different run‐in trials. We also completed a robustness analysis using a parametric bootstrapping method with 1000 iterations. The bootstrap algorithm takes into account variability (at the study level), variability attributed to the selection of studies, and correlation structure attributed to nonindependence of matched pairs. During each iteration, we resampled each RR from a normal distribution with mean of the log(RR) and SD equal to the RR variance to account for the sampling error of each trial RR estimate. For each possible run‐in and non–run‐in match, we calculated the log‐RRR by subtracting the corresponding log‐RR and resampled all pairs with replacement. In this analysis, we calculated the mean, 2.5% quantile, and 97.5% quantile from the 1000 summary estimates of RRRs to obtain a parametric bootstrapping summary estimate and CI.

To address the degree to which our matching ameliorates confounding, we completed an analysis without individual trial matching (ie, comparison of meta‐analytic estimate of the 2 groups of trials). First, we calculated the summary run‐in and non–run‐in RR estimates using random‐effects meta‐analysis, and then we calculated the log(run‐in RR)−log(non–run‐in RR) to obtain an RRR. We then calculated the corresponding E‐value^28^ for the upper bound of CI: UL*=1/UL and E‐value=UL*+sqrt(UL*×[UL*−1]).

We additionally performed a random‐effects meta‐regression analysis with log‐transformed treatment effect as our outcome and run‐in status as our predictor variable, with univariate and multivariable adjusting for mean age, sex, trial sample size, duration of follow‐up, primary versus secondary prevention, and year of publication.

To explore differences in clinical event rates between run‐in and non–run‐in trial populations, we compared incidence rates of clinical events and loss to follow‐up in the control groups of trials using an inverse variance weighting meta‐analysis to give higher importance to studies of larger sample sizes.

### Assessment of the Quality of the Studies: Risk of Bias

We used the Cochrane Risk of Bias Tool to assess methodological quality of eligible trials including random sequence generation, allocation concealment, blinding of participants and health care personnel, blinded outcome assessment, completeness of outcome data, evidence of selective reporting, and other biases.^29^ Risk‐of‐bias assessments were performed independently by 2 reviewers, and disagreements resolved by a third reviewer.

## RESULTS

### Comparison of Characteristics of Run‐In and Non–Run‐In Trials

From the 11 identified systematic reviews, a total of 177 randomized clinical trials were identified, with 66 eligible run‐in trials and 111 eligible non–run‐in trials with a total sample size of 668 901 patients (Table S1). Table 1 describes the characteristics of the run‐in and non–run‐in studies included in our primary cardiovascular composite outcome analysis with 32 run‐in trials and 76 non–run‐in trials (ie, best matches). The majority (88%) were parallel group trials. Run‐in trials had older populations (mean±SD age, 64.5±6.28 versus 61.0±6.20; *P*=0.01) and larger samples sizes, with the mean number of participants in the run‐in trials (mean±SD sample size, 6604±7243) significantly larger than in the non–run‐in trials (mean±SD sample size, 3471±4036; *P*=0.03). Overall, 78% of run‐in trials were reported in high‐impact journals compared with 59% of non–run‐in trials (*P*=0.09). Risk‐of‐bias comparisons were similar between run‐in and non–run‐in studies (Table 1 and Figure S4). The mean±SD duration of follow‐up were similar: 42.8±17.5 months versus 42.7±17.7 months (*P*=0.97). A meta‐analysis of the matched run‐in and non–run‐in trials found no significant difference for loss to follow‐up (RRR, 1.04 [95% CI, 0.84–1.30]). Differences between placebo run‐in and active run‐in studies are included in Table S3. Active run‐in was more commonly performed in antihypertensive studies than in studies of lipid‐lowering or glucose‐lowering agents (*P*=0.05).

**Table 1:** Characteristics of Run‐In and Non–Run‐In Trials Matched by Cardiovascular Composite Outcome

	Run‐in (n=32)	Non–run‐in n=76)	* *P* value
Year of publication			0.37
Before 1990	1 (3.12)	5 (6.58)	
1990–2000	8 (25.0)	9 (11.8)	
2001–2010	13 (40.6)	32 (42.1)	
2011–2020	10 (31.2)	30 (39.5)	
Experimental design			0.52
Factorial	5 (15.6)	8 (10.5)	
Parallel	27 (84.4)	68 (89.5)	
Study characteristics			1.0
Blood pressure–lowering agent	16 (53.3)	38 (52.1)	
Glucose‐lowering agent	1 (3.33)	2 (2.74)	
Lipid‐lowering agent	13 (43.3)	33 (45.2)	
Prevention type			0.89
Primary	18 (56.2)	40 (52.6)	
Secondary	14 (43.8)	36 (47.4)	
Published in high‐impact journal			0.1
No	7 (21.9)	31 (40.8)	
Yes	25 (78.1)	45 (59.2)	
Industry supported			1.0
No	1 (3.12)	4 (5.26)	
Yes	31 (96.9)	70 (92.1)	
Not reported	0 (0.00)	2 (2.63)	
Age, y	64.9±6.28	61.0±6.20	0.01
Composite primary outcome			0.09
No	10 (31.2)	39 (51.3)	
Yes	22 (68.8)	37 (48.7)	
Number of patients randomly assigned	6604±7243	3471±4036	0.03
Duration of follow‐up, mo	42.8±17.5	42.7±17.7	0.97
Total lost to follow‐up, %	1.80±3.84	1.26±2.65	0.55
Random sequence generation			0.04
Low	21 (65.6)	56 (73.7)	
Unclear	8 (25.0)	20 (26.3)	
High	3 (9.38)	0 (0.00)	
Allocation concealment			0.4
Low	21 (65.6)	53 (69.7)	
Unclear	9 (28.1)	22 (28.9)	
High	2 (6.25)	1 (1.32)	
Blinding of participants and personnel			0.15
Low	30 (93.8)	59 (77.6)	
Unclear	0 (0.00)	5 (6.58)	
High	2 (6.25)	12 (15.8)	
Blinding outcome assessors			0.69
Low	26 (81.2)	65 (85.5)	
Unclear	6 (18.8)	10 (13.2)	
High	0 (0.00)	1 (1.32)	
Selective reporting			0.86
Low	30 (93.8)	68 (89.5)	
Unclear	1 (3.12)	2 (2.63)	
High	1 (3.12)	6 (7.89)	
Other bias			0.21
Low	25 (78.1)	67 (88.2)	
Unclear	0 (0.00)	1 (1.32)	
High	7 (21.9)	8 (10.5)	

Data are provided as mean±SD or number (percentage).

* Between‐group significant differences were calculated using *t* tests and Pearson chi‐squared tests for categorical variables.

### Cardiovascular Composite Outcome

**Figure 1:**
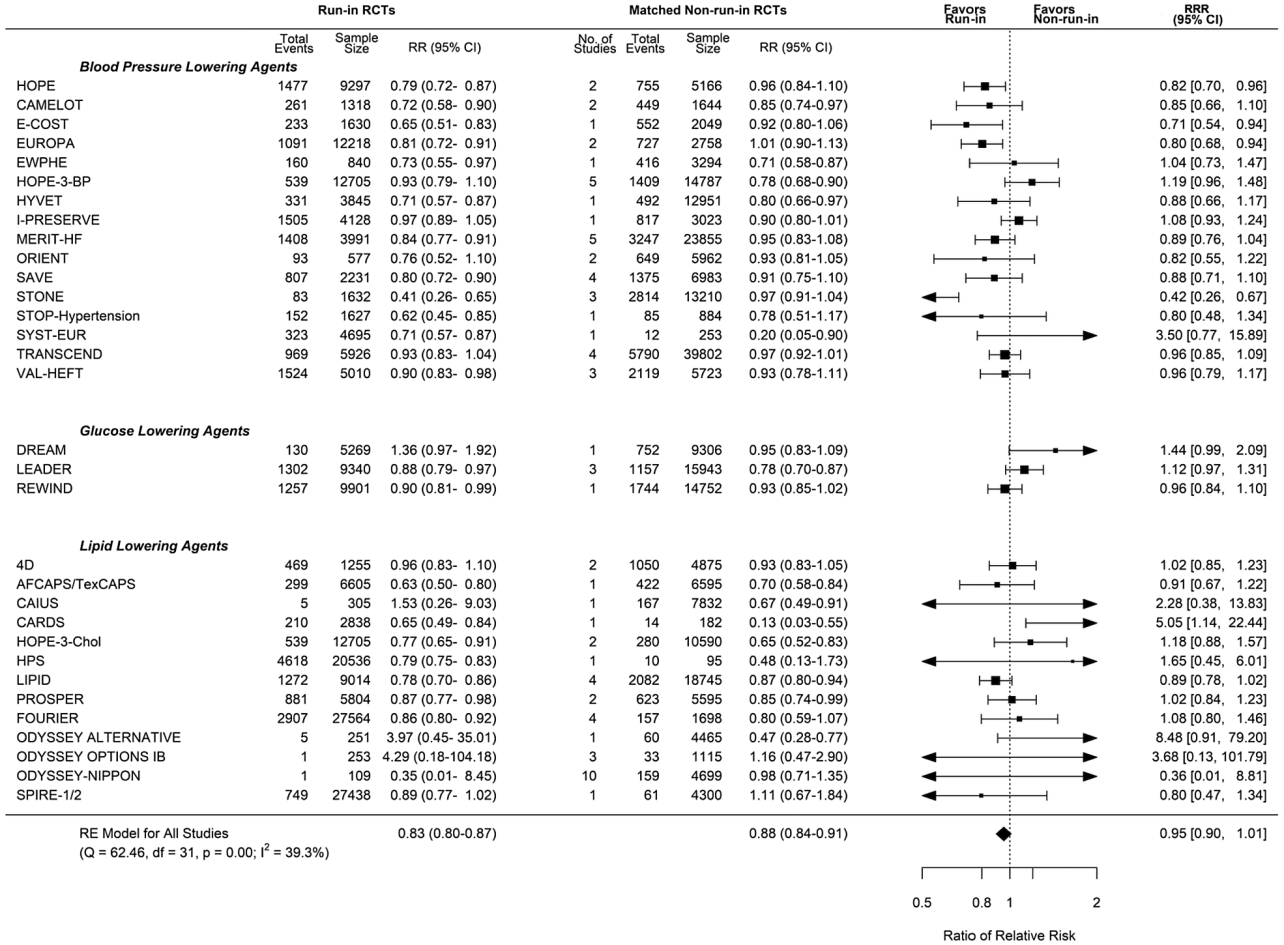
Pooled ratio of relative risks (RRR) and 95% CIs for run‐in versus non–run‐in randomized clinical trials (RCTs): cardiovascular composite. Single best population, intervention, control, and outcome score analysis. Pooled ratio of relative risks (RRR) for matched run‐in and non–run‐in RCTs that reported a cardiovascular composite outcome. “No. of Studies” refers to the number of non–run‐in trials that have been matched to each individual run‐in trial. The size of the data markers indicates the weight of the comparison in the meta‐analysis. A summary estimate <1 represents an exaggerated treatment effect of run‐in. df indicates degrees of freedom; I^2^, degree of heterogeneity; Q, Cochran’s Q; RE, random‐effects meta‐analysis; and RR, relative risk.

A total of 32 run‐in trials matched with ≥1 non–run‐in trials, of which 15 were matched with multiple non–run‐in trials. There was no significant difference in treatment effect for cardiovascular events between run‐in trials (RR, 0.83 [95% CI, 0.80–0.87]) compared with non–run‐in trials (RR, 0.88 [95% CI, 0.84–0.91]; RRR, 0.95 [95% CI, 0.90–1.01]; Figure 1). Sensitivity analyses including multiple‐matches analysis and parametric bootstrapping analysis increased the precision of our estimate but were not statistically significant (Table 2). Our latent confounding sensitivity analysis suggested that an unmeasured confounder, associated with run‐in and RR, with a risk ratio of 1.16 could explain the upper confidence limit, but weaker confounding could not. Post hoc exploratory univariable meta‐regression analysis showed that on average a run‐in period was associated with a statistically significant difference in treatment effects (RRR, 0.94 [95% CI, 0.90–0.99]) for cardiovascular composite outcome. This was not statistically significant on multivariable meta‐regression analysis adjusting for mean age, sex, trial sample size, duration of follow‐up, primary versus secondary prevention, and year of publication (RRR, 0.95 [95% CI, 0.90–1]). Sensitivity analysis using fixed‐effects meta‐analysis of the non–run‐in trials did not alter our findings (RRR, 0.95 [95% CI, 0.90–1.01]).

**Table 2:** Summary Estimates of Relative Treatment Estimates

Outcome	Run‐in summary estimate	Non–run‐in summary estimate	Single best match	Univariable meta‐regression	Multivariable meta‐regression	Multiple matches	Parametric bootstrapping
	Relative risk (95% CI)	Relative risk (95% CI)	Ratio of relative risk (95% CI)	Ratio of relative risk (95% CI)	Ratio of relative risk (95% CI)	Ratio of relative risk (95% CI)	Ratio of relative risk (95% CI)
Composite CVD outcome	0.83 (0.80–0.87)	0.88 (0.84–0.91)	0.95 (0.90–1.01)	0.94 (0.90–0.99)	0.95 (0.90–1.00)	0.97 (0.93–1.02)	0.98 (0.94–1.02)
All‐cause mortality	0.89 (0.86–0.93)	0.95 (0.92–0.98)	0.97 (0.91–1.03)	0.95 (0.90–1.00)	0.94 (0.89–1.00)	0.98 (0.95–1.01)	0.98 (0.93–1.03)
Adverse events	1.22 (1.06–1.40)	1.19 (1.06–1.34)	1.05 (0.85–1.29)	0.90 (0.73–1.12)	0.86 (0.68–1.07)	1.03 (0.87–1.21)	0.93 (0.74–1.09)
Nonfatal myocardial infarction	0.82 (0.75–0.89)	0.83 (0.75–0.92)	1.01 (0.85–1.19)	0.95 (0.85–1.07)	0.99 (0.88–1.12)	0.99 (0.90–1.08)	0.98 (0.88–1.11)
Nonfatal stroke	0.77 (0.72–0.83)	0.83 (0.75–0.93)	1.00 (0.87–1.16)	0.92 (0.84–1.02)	0.98 (0.87–1.10)	0.99 (0.89–1.11)	0.97 (0.90–1.06)

Multivariable meta‐regression adjusted for mean age, prevention type, duration of follow‐up (months), sex, publication year, and sample size. CVD indicates cardiovascular disease.

### All‐Cause Mortality Outcome

**Figure 2:**
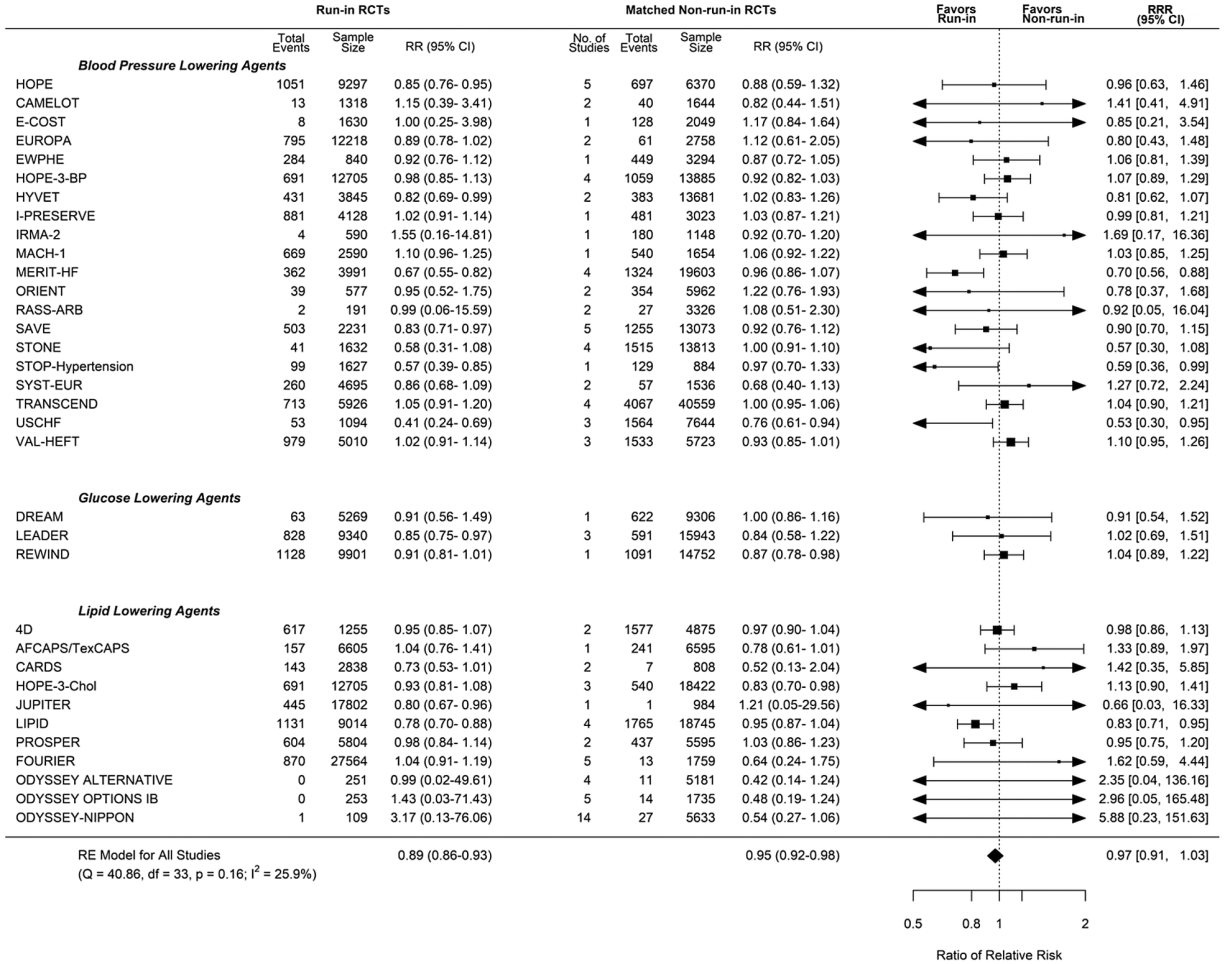
Pooled ratio of relative risks and 95% CIs for run‐in versus non–run‐in randomized clinical trials (RCTs): all‐cause mortality. Single best population, intervention, control, and outcome score analysis. Pooled ratio of relative risks for matched run‐in and non–run‐in RCTs that reported mortality outcomes. “No of Studies” refers to the number of non–run‐in trials that have been matched to each individual run‐in trial. The size of the data markers indicates the weight of the comparison in the meta‐analysis. A summary estimate <1 represents an exaggerated treatment effect of run‐in. df indicates degrees of freedom; I^2^, degree of heterogeneity; Q, Cochran’s Q; RE, random‐effects meta‐analysis; and RR, relative risk.

A total of 34 run‐in trials matched with ≥1 run‐in trials, of which 24 were matched with multiple non–run‐in trials. There was no difference in mortality incidence rate in control groups of run‐in studies (24.7 per 1000 person‐years) compared with non–run‐in studies (27.9 per 1000 person‐years; incidence difference, −5.3 [95% CI, −24.69 to 14.09]; Table S4). There was no significant difference in treatment effect for mortality in run‐in trials (RR, 0.89 [95% CI, 0.86–0.93]) compared with non–run‐in trials (RR, 0.95 [95% CI, 0.92–0.98]; RRR, 0.97 [95% CI, 0.91–1.03]; Figure 2). Sensitivity analyses including multiple‐matches analysis, bootstrapping analysis, or meta‐regression did not alter the findings (Table 2).

### Adverse Events Leading to Permanent Medication Discontinuation Outcome

**Figure 3:**
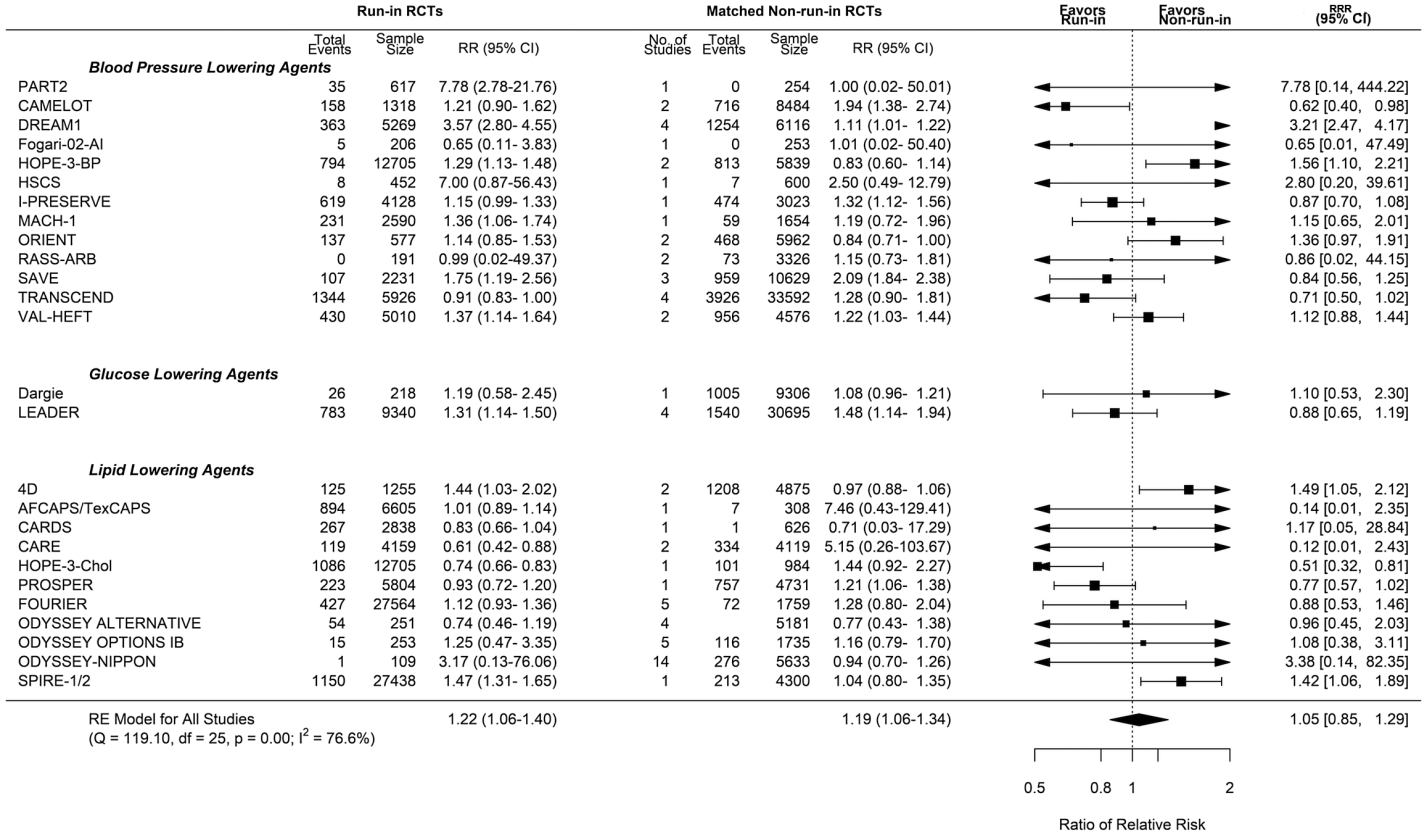
Pooled ratio of relative risks and 95% CIs for run‐in versus non–run‐in randomized clinical trials (RCTs): adverse events leading to medication discontinuation. Single best population, intervention, control, and outcome score analysis. Pooled ratio of relative risks for matched run‐in and non–run‐in RCTs that reported adverse events leading to discontinuation of study medication. “No. of Studies” refers to the number of non–run‐in trials that have been matched to each individual run‐in trial. The size of the data markers indicates the weight of the comparison in the meta‐analysis. A summary estimate <1 represents an exaggerated treatment effect of run‐in. df indicates degrees of freedom; I^2^, degree of heterogeneity; Q, Cochran’s Q; RE, random‐effects meta‐analysis; and RR, relative risk.

A total of 26 run‐in trials were matched with non–run‐in trials, of which 15 were matched with multiple non–run‐in trials. There was no statistically significant difference in the incidence rate of adverse events leading to permanent medication discontinuation among control groups between run‐in studies (12.8 per 1000 person‐years) and non–run‐in studies (34.9 per 1000 person‐years; incidence difference, −9.49 [95% CI, −28.1 to 9.1]; Table S4). There was no significant difference in adverse events leading to permanent discontinuation between run‐in trials (RR, 1.22 [95% CI, 1.06–1.40]) and non–run‐in trials (RR, 1.19 [95% CI, 1.06–1.34]; RRR, 1.05 [95% CI, 0.85–1.21]; Figure 3). Sensitivity analyses including multiple‐matches analysis, bootstrapping analysis, or meta‐regression did not alter the findings (Table 2).

### Nonfatal Myocardial Infarction and Nonfatal Stroke Outcome

A random‐effects meta‐analysis of the RRRs between run‐in and matched non–run‐in studies showed no statistically significant difference in treatment effects for nonfatal myocardial infarction (RRR, 1.01 [95% CI, 0.85–1.19]) or nonfatal stroke (RRR, 1.00 [95% CI, 0.87–1.16]; Table 2). Sensitivity analyses including multiple‐matches analysis, bootstrapping analysis, or meta‐regression did not alter the findings (Table 2).

### Quality of Reporting of Run‐In

Authors reported the reason for a run‐in design in 48 of 66 trials (72%; Table S5). Of the run‐in trials, 38 (58%) reported the absolute number of exclusions during the run‐in period. Individual reasons for participant exclusions during run‐in was reported in 30 run‐in trials (45%), with 20 trials clearly reporting the total number of exclusions during run‐in attributed to adverse events during the run‐in period.

Placebo run‐in alone was conducted in 42 trials, active run‐in alone was conducted in 14 trials, and 9 trials had both an active and placebo run‐in period. In 1 trial it was unclear whether placebo or active run‐in was used. Loss to follow‐up during the run‐in period was reported in 38 trials (58%). The median reported duration of placebo run‐in was 4 weeks (range, 1 week–4 months). The median reported duration of active run‐in was 4 weeks (range, 1 day–3 months). The median reported percentage of patients excluded during active run‐in was 13.5%. The median reported percentage of patients excluded during placebo run‐in was 12.3%.

## DISCUSSION

We did not find a significant difference in the relative treatment effect of run‐in trials compared with matched non–run‐in trials for several clinical outcomes—a composite of cardiovascular outcomes, mortality, adverse events, nonfatal myocardial infarction, and nonfatal stroke. We also failed to identify differences in adverse events leading to medication withdrawal or the proportion of participants lost to follow‐up, which are expected advantages of using run‐in periods in randomized clinical trials.

Our central hypothesis was that treatment effects reported in clinical trials that used run‐in may be larger than those reported in trials that did not use a run‐in period, which may have implications for guideline recommendations. The rationale to suspect differences is based on the contention that populations included in trials that used run‐in would differ from those in non–run‐in trials, as suspected and reported by previous researchers.^6^, ^8^ In previous meta‐analyses, comparisons of run‐in and non–run‐in trials have been confined to smaller groups of trials.^30^ In our larger analyses, the lower bound of the CI for RRR ranged from 0.90 to 0.94 based on approaches of bootstrapping analysis and meta‐regression, meaning that a treatment difference in this range is still possible but unlikely. The only analysis reporting a significant association was our univariable meta‐regression, which reported a 6% difference in RR associated with run‐in but was not significant on adjusting for factors that differed between run‐in and non–run‐in trials (eg, sample size). By comparison, a previous analysis evaluating the effect of early stopping of clinical trials reported an RRR of 30%,^31^ resulting in revision to the Grading of Recommendations, Assessment, Development, and Evaluations system for guideline recommendations, with rating down quality because of risk of bias for trials that stopped early because of efficacy.^32^ In our analyses, we did not find a significant difference in the RRR between trials with and without run‐in periods, and our findings suggest that if a difference does exist, it is of a magnitude that should not affect the quality of evidence assessments in guideline recommendations or influence physician prescribing patterns.

We expected to find differences in clinical event rates, adverse events leading to drug discontinuation, and loss to follow‐up given that a run‐in period is intended to exclude participants at higher risk of adverse events and nonadherence with trial protocol. Although adverse events were numerically more common in non–run‐in trials, it was not statistically significant, and the RR of adverse events was similar in both trial groups. These observations provide empirical evidence that relative adverse treatment effects derived from run‐in trials are unlikely to be biased compared with nonuse of a run‐in period. However, our findings would also appear to challenge whether a run‐in period is truly associated with the intended effect on trial populations given that we did not observe significant differences in adverse event rates or loss to follow‐up. Moreover, the similar rates of mortality and cardiovascular events does not support a major selection bias in cardiovascular risk in populations included in trials employing a run‐in period. Against this observation, individual trial analyses of populations excluded during run‐in report differences in risk and event rates, which may reflect different effects depending on the clinical population. Of note, our analysis may have been underpowered to show differences in control incidence rates, which may become more apparent and significant in a larger study.^1^

Reviews of the reporting quality of published randomized clinical trials have shown inconsistent quality of reporting, and some trials failed to record participant flow clearly, which is particularly common in the stages before randomization.^33^ We also found inconsistencies in reporting the proportion of patients excluded during the run‐in period and the reasons for their exclusion. Our findings are in keeping with similar findings for randomization, blinding, and attrition.^34^, ^35^, ^36^ Previous research has found that when improved reporting standards have been adopted, such as the Consolidated Standards of Reporting Trials checklist for randomized clinical trials, this has been associated with improved reporting of randomized clinical trials.^37^, ^38^ It would appear reasonable that the Consolidated Standards of Reporting Trials statement include requirements for standardized descriptions of the run‐in periods.

The strengths of our study include a robust methodology with use of a matching strategy, resulting in a comprehensive meta‐analytic examination of run‐in periods among cardiovascular prevention trials. We included a large number of randomized clinical trials (n=177) that were systematically and independently reviewed across a range of journals and covering a broad period from 1990 to 2019. We chose cause‐specific clinical outcomes that can be classified with reasonable reproducibility, which reduces the risk of ascertainment bias.^39^, ^40^ We also completed a range of secondary analyses completed for each clinical outcome.^41^, ^42^

Limitations of our study include the number of clinical trials included in our analyses and the fact that our analyses were restricted to cardiovascular prevention trials of medicines, meaning that these findings may not be extended to other populations or interventions. However, confining our analyses to 1 clinical area may also be considered a strength as it reduced heterogeneity in clinical trial design, population, and outcome measures. In some of our analyses, a low proportion of trials were included, such as loss to follow‐up. We did not include an outcome for all adverse events because of inconsistent reporting, and we were therefore unable to report on the association of run‐in period with rates of all adverse events. We do report the more valid and reliable outcome of adverse event leading to drug discontinuation. Finally, although our findings have implications to all clinical trials employing a run‐in period, they are only directly relevant to cardiovascular prevention trials of established preventive medications.^43^

## CONCLUSIONS

In conclusion, the use of a run‐in period was not associated with a significant difference in the magnitude of treatment effect among randomized controlled trials evaluating medications to prevent cardiovascular events.

## Sources of Funding

Dr Judge was supported by the Irish Clinical Academic Training Programme, the Wellcome Trust, the Health Research Board (Grant 203930/B/16/Z), the Health Service Executive, National Doctors Training and Planning, and the Health and Social Care, Research and Development Division, Northern Ireland. Dr O’Donnell was supported by the European Research Council (Clarifying Optimal Sodium Intake Porject Grant 640580). The funding sources had no role in the design and conduct of the study; the collection, management, analysis, and interpretation of the data; the preparation, review, or approval of the manuscript; or the decision to submit the manuscript for publication.

## Disclosures

None.

## Supporting information

Tables S1–S5
Figures S1–S4
