# Supplementary material for: Effect of a Run‐In Period on Estimated Treatment Effects in Cardiovascular Randomized Clinical Trials: A Meta‐Analytic Review
Source: J Am Heart Assoc. 2022 Oct 17;11(20):e023061. doi: 10.1161/JAHA.121.023061 (PMC9673681; doi:10.1161/JAHA.121.023061)

Supplemental Material

Table S1. Source Systematic Reviews.

**Anti-hypertensive Therapies**		
Blood pressure lowering for prevention of cardiovascular disease and death: a systematic review and meta-analysis	Ettehad D, Emdin CA, Kiran A, et al	Lancet. 2016; 387(10022):957-967.
Association of Blood Pressure Lowering With Mortality and Cardiovascular Disease Across Blood Pressure Levels: A Systematic Review and Meta-analysis	Brunstrom M, Carlberg B	*JAMA internal medicine. 2018;178(1):28-36.*
Blood pressure-lowering drugs and secondary prevention of cardiovascular disease: systematic review and meta-analysis.	Xie W, Zheng F, Evangelou E, et al	Journal of hypertension. 2018;36(6):1256-1265.
Antihypertensive treatment and secondary prevention of cardiovascular disease events among persons without hypertension: a meta-analysis	Thompson AM, Hu T, Eshelbrenner CL, at al.	*Jama. 2011;305(9):913-922*
The effects of blood pressure reduction and of different blood pressure-lowering regimens on major cardiovascular events according to baseline blood pressure: meta-analysis of randomized trials	Czernichow S, Zanchetti A, Turnbull F, et al.	Journal of hypertension. 2011;29(1):4-16.
**Lipid Lowering Therapies**		
Statins for Prevention of Cardiovascular Disease in Adults: Evidence Report and Systematic Review for the US Preventive Services Task Force	Chou R, Dana T, Blazina I, et al.	Jama. 2016;316(19):2008-2024.
Effect of statins and non-statin LDL-lowering medications on cardiovascular outcomes in secondary prevention: a meta-analysis of randomized trials.	Koskinas KC, Siontis GCM, Piccolo R, et al.	*European heart journal. 2018;39(14):1172-1180.*
Efficacy and safety of more intensive lowering of LDL cholesterol: a meta-analysis of data from 170,000 participants in 26 randomised trials.	Baigent C, Blackwell L, Emberson J, et al.	Lancet. 2010 ;376(9753):1670-1681.
Proprotein convertase subtilisin/kexin 9 inhibitors in reducing cardiovascular outcomes: a systematic review and meta-analysis	Du H, Li X, Su N, *et al*	*Heart Epub ahead of print: doi:10.1136/ heartjnl-2019-314763*
**Glucose Lowering Therapies**		
Cardiovascular, mortality, and kidney outcomes with GLP-1 receptor agonists in patients with type 2 diabetes: a systematic review and meta-analysis of cardiovascular outcome trials	Kristensen S, Rørth R, Jhund P, et al.	Lancet Diabetes Endocrinol
SGLT2 inhibitors for primary and secondary prevention of cardiovascular and renal outcomes in type 2 diabetes: a systematic review and meta-analysis of cardiovascular outcome trials	*Zelniker T, Wiviott S, Raz I, et al.*	*. 2019 Oct;7(10):776-785.*

Table S2. Run-in and Non-run-in Matching Score Considerations.

**Population**<br>• Main considerations: prevention type (primary vs secondary), population summary, mean age, gender match<br>• Studies that compare primary prevention and secondary prevention populations are not a match<br>• Chronic stable secondary prevention populations do not match with studies that include populations who recruit patients with acute events<br>• Inclusion criteria should be similar in age, cardiovascular comorbidity population and inclusion criteria
**Intervention**<br>• Main considerations: drug and dose<br>• Same drugs - scored 3<br>• Same drug class and pharmacologically similar - scored 2<br>• Same drug class but pharmacologically dissimilar - scored 1<br>• Different drug class – scored 0
**Control**<br>• Main considerations: placebo control vs standard treatment.<br>• If both studies are placebo controlled - scored 3<br>• If placebo is compared with standard treatment and an additional medication - scored 2<br>• If placebo is compared with standard treatment - scored 1
**Outcome**<br>• Similar CV Composite/Primary Outcome - scored 3<br>• Report multiple outcomes for mortality/stroke/MI - scored 2<br>• If report one of mortality/stroke/myocardial infarction - scored 1<br>• If report no similar outcome - score 0

Table S3. Comparison of Placebo Run-in and Active Run-in Studies.

	**Placebo Run-in N=42**	**Active Run-in N=14**	**P Value**
Year of Publication:			0.92
Before 1990	2 (4.76%)	0 (0.00%)	
1990-2000	10 (23.8%)	3 (21.4%)	
2001-2010	16 (38.1%)	7 (50.0%)	
2011-2020	14 (33.3%)	4 (28.6%)	
Experimental Design:			0.26
Factorial	2 (4.76%)	2 (14.3%)	
Parallel	40 (95.2%)	12 (85.7%)	
Study Characteristics:			0.05
Blood Pressure Lowering Agent	18 (45.0%)	11 (78.6%)	
Glucose Lowering Agent	6 (15.0%)	2 (14.3%)	
Lipid Lowering Agent	16 (40.0%)	1 (7.14%)	
Prevention type:			0.35
Primary Prevention	26 (61.9%)	6 (42.9%)	
Secondary Prevention	16 (38.1%)	8 (57.1%)	
Published in high impact journal:			0.73
No	13 (31.0%)	3 (21.4%)	
Yes	29 (69.0%)	11 (78.6%)	
Industry Supported:			1.00
No	1 (2.38%)	0 (0.00%)	
Yes	41 (97.6%)	14 (100%)	
Composite Primary Outcome:			0.88
No	18 (42.9%)	7 (50.0%)	
Yes	24 (57.1%)	7 (50.0%)	
Number of patients randomised:	5423 (6937)	5762 (4622)	0.84
Duration of follow-up (Months):	36.6 (15.6)	45.3 (17.3)	0.14
Random Sequence Generation:			0.23
Low	25 (59.5%)	12 (85.7%)	
Unclear	15 (35.7%)	2 (14.3%)	
High	2 (4.76%)	0 (0.00%)	
Allocation Concealment:			0.23
Low	25 (59.5%)	12 (85.7%)	
Unclear	15 (35.7%)	2 (14.3%)	
High	2 (4.76%)	0 (0.00%)	
Blinding of Participants and Personnel:			0.15

	Placebo Run-in N=42	Active Run-in N=14	P Value
Low	41 (97.6%)	12 (85.7%)	
High	1 (2.38%)	2 (14.3%)	
Blinding outcome assessors:			1.0
Low	35 (83.3%)	12 (85.7%)	
Unclear	7 (16.7%)	2 (14.3%)	
Selective Reporting:			0.53
Low	39 (92.9%)	12 (85.7%)	
Unclear	2 (4.76%)	1 (7.14%)	
High	1 (2.38%)	1 (7.14%)	
Other Bias:			0.18
Low	34 (81.0%)	14 (100%)	
High	8 (19.0%)	0 (0.00%)	

Table S4. Comparison of Incidence Rates.

Outcome	Run-in Incidence per 1000 person-years	Non-run-in Incidence per 1000 person-years	Incidence Difference per 1000 person-years
CVD Composite	52.96 (14.48-91.45)	41.81 (25.05-58.56)	6.6 (-35.88 to 49.07)
All-cause Mortality	24.74 (12.58-36.9)	27.92 (14.73-41.11)	-5.3 (-24.69 to 14.09)
Non-fatal Myocardial Infarction	12.71 (7.7-17.72)	12.26 (5.39-19.13)	0.6 (-6.52 to 7.71)
Non-fatal Stroke	9.86 (6.57-13.15)	14.66 (4.87-24.44)	-4.82 (-11.25 to 1.62)
Adverse Events	12.76 (6.55-18.97)	34.93 (18.53-51.33)	-9.49 (-28.07 to 9.1)
Loss to follow-up	10.61 (0.71-20.51)	8.17 (2.07-14.28)	1.53 (-9.9 to 12.95)

Table S5. Characteristics of all Run-in Trials Identified.

Characteristics of Run-In Trials	n=66 (%)
Both Placebo and Active Run-in	9 (13.63)
Placebo Run-In Only	42 (63.63)
Active Run-In Only	14 (21.21)
Type of Run-in Not Reported	1 (1.51)
Reported Reason for Run-in phase	48 (72.72)
Reported total exclusions during Run-In	38 (57.57)
Reported individual reasons for exclusions	30 (45.45)
Reported adverse events during run-in	20 (30.30)
Timing of Active and Placebo Run-in	n=9
Placebo Run-in First	5
Active Run-in First	3
Simultaneous with factorial design	1
Median Duration Active Run-in (Weeks)	4 (1 day - 3-month range)
Median Duration Placebo Run-in (Weeks)	4 (1 week - 4-month range)

Figure S1. Flow Diagram of Included Trials.

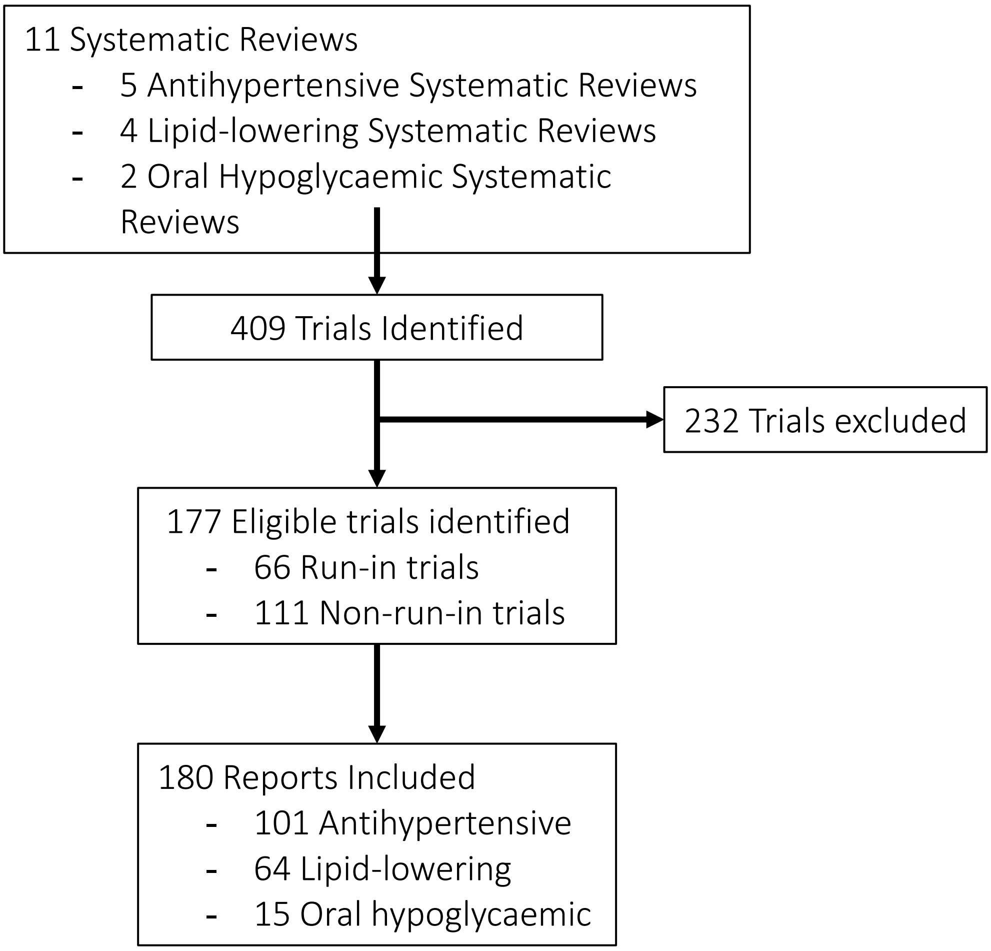

Figure S2. PICO Scoring Steps.

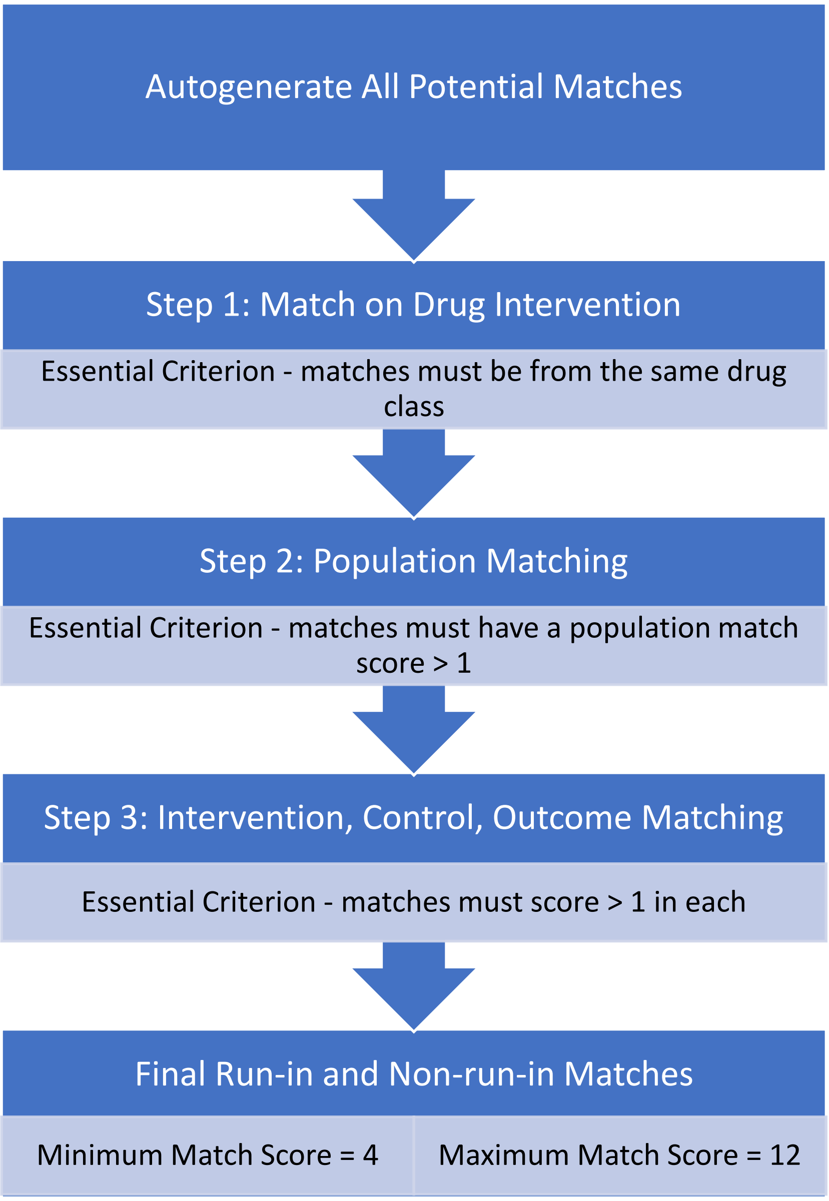

Figure S3. Visual Representation of Different PICO Matching Scenarios.

Scenario 1: Highest PICO scenario
Non-run-in trials were matched to their corresponding highest PICO score run-in trial(s) e.g. Trial A and Trial C with total PICO 12/12.

Run-in Trials	Run-in Participants	Non-run-in Trials	Population /3	Intervention /3	Control /3	Outcome /3	Total /12
Trial A	6000	Trial C	3	3	3	3	12
Trial A	6000	Trial D	2	1	3	3	9
Trial A	6000	Trial E	3	2	2	3	10
~~Trial B~~	~~8000~~	~~Trial C~~	~~3~~	~~3~~	~~2~~	~~3~~	~~11~~
Trial B	8000	Trial F	3	3	3	3	12
Trial B	8000	Trial G	3	1	2	3	9

Scenario 2: Equal PICO scenario
If several trials have equal PICO matches, we selected the trials with the largest non-run-in sample size e.g. Trial B and Trial C with run-in sample size of 8000.

Run-in Trials	Run-in Participants	Non-run-in Trials	Population /3	Intervention /3	Control /3	Outcome /3	Total /12
~~Trial A~~	~~6000~~	~~Trial C~~	~~3~~	~~3~~	~~2~~	~~3~~	~~11~~
Trial A	6000	Trial D	2	1	3	3	9
Trial A	6000	Trial E	3	2	2	3	10
Trial B	8000	Trial C	3	3	2	3	11
Trial B	8000	Trial F	3	3	3	3	12
Trial B	8000	Trial G	3	1	2	3	9

Figure S4. Risk of Bias Overall Summary For Trials included in Cardiovascular Composite Outcome Analysis.

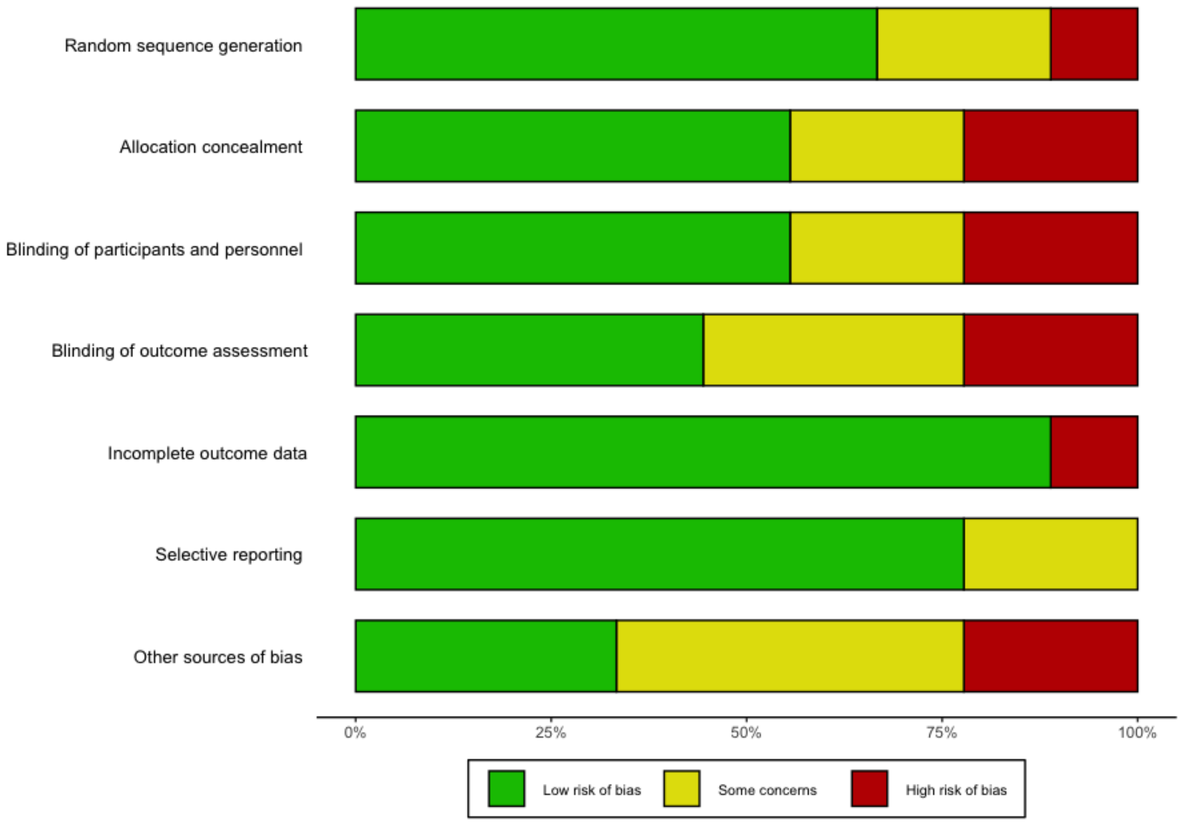

Supplement: Supplementary file 1 — Tables S1–S5 Figures S1–S4 [file JAH3-11-e023061-s001.pdf]
